# Bayesian spatio-temporal modelling of environmental, climatic, and socio-economic influences on malaria in Central Vietnam

**DOI:** 10.1186/s12936-024-05074-y

**Published:** 2024-08-24

**Authors:** Le Thanh Tam, Kavin Thinkhamrop, Sutas Suttiprapa, Archie C. A. Clements, Kinley Wangdi, Apiporn T. Suwannatrai

**Affiliations:** 1 Department of Tropical Medicine, Faculty of Medicine, Khon Kaen University, Khon Kaen, Thailand; 2 Department of Epidemiology, Institute of Malariology, Parasitology, and Entomology Quy Nhon, Quy Nhon, Binh Dinh Vietnam; 3 Health and Epidemiology Geoinformatics Research (HEGER), Faculty of Public Health, Khon Kaen University, Khon Kaen, Thailand; 4 Queen’s University Belfast, Belfast, Northern Ireland UK; 5 HEAL Global Research Centre, Health Research Institute, University of Canberra, Canberra, ACT 2617 Australia; 6 National Centre for Epidemiology and Population Health, College of Health and Medicine, Australian National University, Canberra, Australia; 7 Department of Parasitology, Faculty of Medicine, Khon Kaen University, Khon Kaen, Thailand

**Keywords:** Vietnam, Bayesian, Spatio-temporal, Malaria, *Plasmodium*, Vector-borne disease

## Abstract

**Background:**

Despite the successful efforts in controlling malaria in Vietnam, the disease remains a significant health concern, particularly in Central Vietnam. This study aimed to assess correlations between environmental, climatic, and socio-economic factors in the district with malaria cases.

**Methods:**

The study was conducted in 15 provinces in Central Vietnam from January 2018 to December 2022. Monthly malaria cases were obtained from the Institute of Malariology, Parasitology, and Entomology Quy Nhon, Vietnam. Environmental, climatic, and socio-economic data were retrieved using a Google Earth Engine script. A multivariable Zero-inflated Poisson regression was undertaken using a Bayesian framework with spatial and spatiotemporal random effects with a conditional autoregressive prior structure. The posterior random effects were estimated using Bayesian Markov Chain Monte Carlo simulation with Gibbs sampling.

**Results:**

There was a total of 5,985 *Plasmodium falciparum* and 2,623 *Plasmodium vivax* cases during the study period. *Plasmodium falciparum* risk increased by five times (95% credible interval [CrI] 4.37, 6.74) for each 1-unit increase of normalized difference vegetation index (NDVI) without lag and by 8% (95% CrI 7%, 9%) for every 1ºC increase in maximum temperature (TMAX) at a 6-month lag. While a decrease in risk of 1% (95% CrI 0%, 1%) for a 1 mm increase in precipitation with a 6-month lag was observed. A 1-unit increase in NDVI at a 1-month lag was associated with a four-fold increase (95% CrI 2.95, 4.90) in risk of *P. vivax*. In addition, the risk increased by 6% (95% CrI 5%, 7%) and 3% (95% CrI 1%, 5%) for each 1ºC increase in land surface temperature during daytime with a 6-month lag and TMAX at a 4-month lag, respectively. Spatial analysis showed a higher mean malaria risk of both species in the Central Highlands and southeast parts of Central Vietnam and a lower risk in the northern and north-western areas.

**Conclusion:**

Identification of environmental, climatic, and socio-economic risk factors and spatial malaria clusters are crucial for designing adaptive strategies to maximize the impact of limited public health resources toward eliminating malaria in Vietnam.

**Supplementary Information:**

The online version contains supplementary material available at 10.1186/s12936-024-05074-y.

## Background

Malaria, a vector-borne disease, continues to pose a significant public health challenge, affecting millions of people worldwide [1]. Despite the successful efforts in controlling malaria in Vietnam, some provinces situated in the Central Highlands region of the country have reported an increase in malaria cases in 2021. Central Vietnam, with its diverse geography and complex environmental dynamics, is one such region where malaria transmission remains a concern [2, 3]. This resurgence can be attributed to several factors including poor accessibility as a result of being mountainous and forested, rendering control measures challenging [4]. Additionally, forest-related activities of the population [2] and poverty [5] are other social factors contributing to continuing malaria transmission in the region. Finally, the presence of the endophagic and anthropophilic vector *Anopheles dirus* [6], makes control measures difficult.

Malaria transmission is not solely determined by the presence of the *Plasmodium* parasite and its *Anopheles* mosquito vectors; it is also intricately linked to a complex interplay of factors. These include temperature, rainfall, land cover, human population density, and socio-economic conditions, which influence the spatial distribution and temporal intensity of malaria cases [7–12]. Temperature impacts mosquito populations, their biting rates, and the development of the malaria parasite within them [13, 14]. Temperature also affects the sporogonic cycle of these mosquitoes [15]. Increased rainfall can create more aquatic habitats, which support the growth of mosquito vectors and increase malaria transmission. Heavy rains, however, can have a negative effect on malaria transmission by killing mosquito larvae and pupae [16]. In water-limited environments, the temporal pattern of rainfall within a season affects the persistence of standing water, which in turn influences mosquito population dynamics [9].

In addition to climatic factors, environmental factors also play an equally important role in the spread of malaria. These factors include normalized difference vegetation index (NDVI), normalized difference water index (NDWI), and land surface temperature (LST). NDVI is crucial for monitoring vegetation that influences *Anopheles* density and malaria transmission, showing strong correlations with malaria incidence rates in Yunnan Province, China [17]. High NDVI values are linked to increased malaria rates, particularly noticeable during the rainy season due to its impact on *Anopheles* density [17, 18]. Also, NDVI changes related to land cover shifts, such as forest to non-forest, correlate with malaria case fluctuations [19]. NDWI helps identify potential mosquito breeding grounds by indicating higher water content areas, though it's indirectly related to malaria. LST is directly associated with malaria transmission, affecting mosquito development and parasite growth, with higher LST areas experiencing more malaria [20, 21]. Nighttime lights (NTL) reflect human activity and urbanization, attracting mosquitoes and potentially increasing malaria transmission by altering mosquito behaviour and extending human activity into the night [22, 23].

The impact of environmental, climatic and socio-economic factors on malaria transmission often involves time lags, reflecting the life cycles of both mosquito vectors and *Plasmodium* parasites [24, 25]. These lag effects are crucial for understanding and predicting malaria incidence patterns [26]. Rainfall typically affects malaria transmission with a lag of 1–2 months [27]. This delay accounts for mosquito breeding, larval development, and the parasite's incubation period in both mosquitoes and humans [28]. Temperature changes can influence malaria incidence with longer lags, often spanning several months [29]. These extended lags reflect the time needed for temperature to affect mosquito population dynamics, parasite development rates, and subsequent changes in human infection patterns [30]. Vegetation changes, measured by NDVI, may impact malaria cases with lags of 1–3 months, affecting mosquito habitats [31]. Socio-economic factors, such as urbanization (often proxied by NTL), can have even longer lag effects, sometimes up to 6 months, as they influence human settlement patterns and vector ecology [32, 33].

Bayesian spatio-temporal analysis is an advanced and comprehensive methodological approach for modelling complex disease patterns, such as those observed in malaria transmission [34, 35]. This analytical framework offers several significant advantages in epidemiological research [36, 37]. It facilitates the integration of both spatial and temporal components of disease transmission, allowing for a more nuanced understanding of epidemiological dynamics [38]. The method accommodates the incorporation of diverse data types, including environmental, climatic, and socio-economic factors, thereby providing a more comprehensive elucidation of disease dynamics [39]. It presents a flexible framework for quantifying prediction uncertainty, which is crucial for informed decision-making in public health interventions [38, 39]. While some Bayesian methods utilize Markov chain Monte Carlo (MCMC) techniques, others employ alternative approaches such as variational inference or approximate Bayesian computation, depending on the specific model requirements and computational constraints [38–40]. The application of these methods is particularly pertinent in the context of Central Vietnam due to several region-specific factors. The area's heterogeneous topography, encompassing coastal regions and highland areas, engenders microclimates that can significantly influence malaria transmission patterns [41–44]. The region is characterized by complex seasonal climatic variations, which can be effectively captured and analysed through spatio-temporal models [45, 46]. Significant socio-economic disparities exist within the region, including varying levels of healthcare accessibility and economic development [47–50]. These factors may impact malaria transmission in ways that can be elucidated through Bayesian analysis. Furthermore, the presence of forest-fringe areas and associated anthropogenic activities in Central Vietnam creates unique spatial patterns of malaria risk that can be effectively modelled using Bayesian spatio-temporal approaches [2, 51].

Despite the potential benefits of this analytical approach, to date, there has been a paucity of comprehensive studies in Vietnam employing Bayesian spatio-temporal analysis that incorporate environmental, climatic, and socio-economic factors as covariates to analyze their association with malaria transmission [34, 52]. This gap in the literature underscores the need for further research utilizing these advanced statistical methods to better understand and predict malaria transmission patterns in this region [53].

Understanding the spatio-temporal patterns of malaria clusters in this region is essential for developing targeted and effective control strategies. This study was undertaken to understand the role of local environmental factors in malaria transmission. Additional aim was to investigate the spatial and temporal patterns of malaria in Central Vietnam.

## Methods

### Study site

**Fig. 1:**
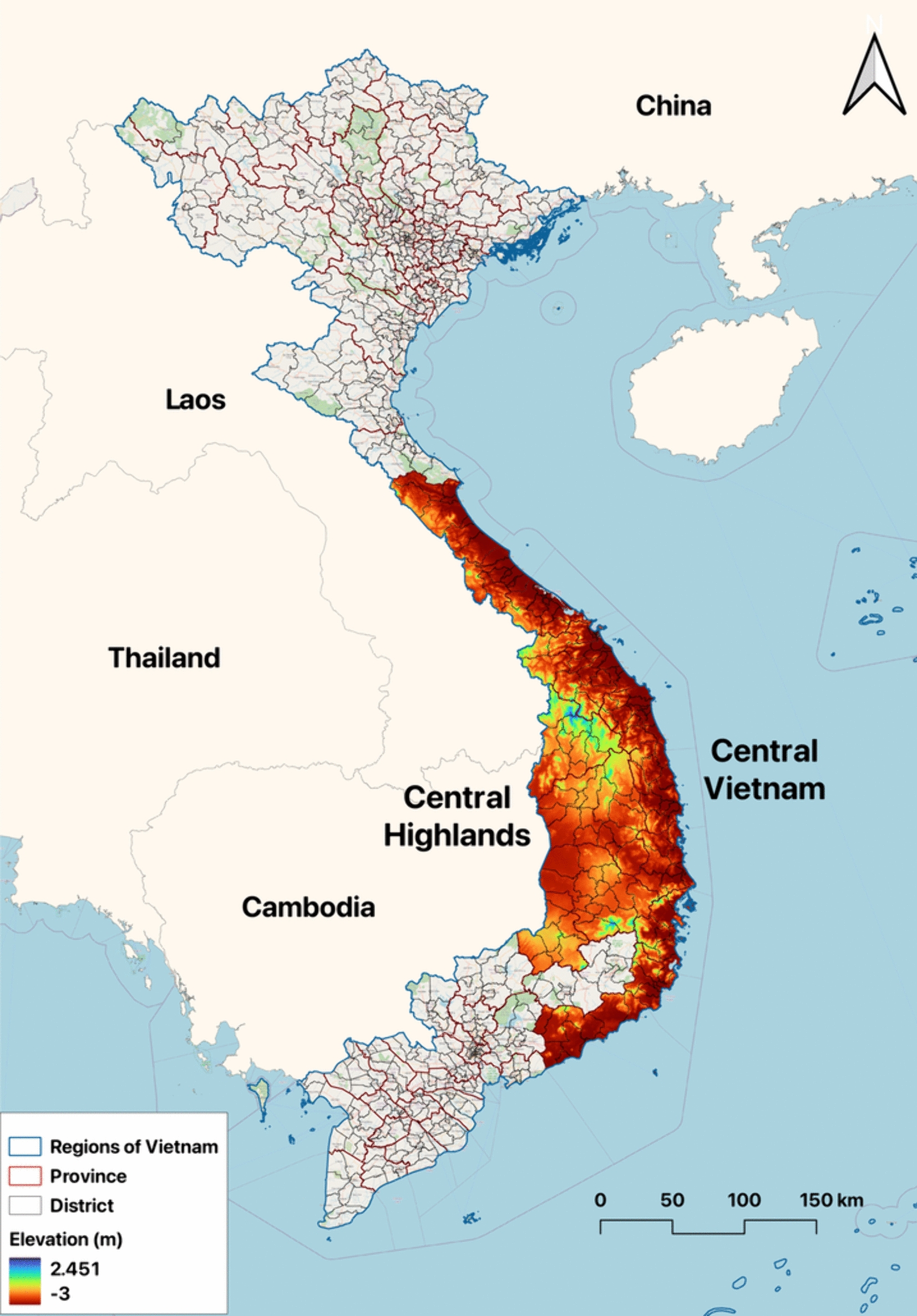
Map of study site with administrative boundaries, neighbouring countries, and altitude

The study was conducted in 15 provinces in Central Vietnam, which consists of 161 districts, and 2,267 communes. In 2019, it had an estimated population of 17.9 million [54]. A map of Vietnam's administrative boundaries and neighbouring countries was created in Quantum GIS (QGIS) version 3.30 ‘s-Hertogenbosch [55] using shape files from the Database of Global Administrative Areas (GADM), version 4.1, available at https://www.gadm.org. (Fig. 1).

Central Vietnam is geographically delineated by the Northwest region, the Red River Delta to the North, and the Southeast region to the South. It is bordered by Laos and Cambodia to the West and the East Sea to the East. The topography of the Central Region is characterized by three primary regions: the North Central Coast, the Central Highlands, and the South Central Coast.

### Data source

#### Malaria cases

Monthly reports of confirmed malaria cases, categorized by species and district, from January 2018 to December 2022, sourced from Institute of Malariology, Parasitology, and Entomology (IMPE) Quy Nhon, Vietnam were obtained. A confirmed malaria case is defined as a malaria case (or infection) in which the parasite has been detected in a diagnostic test, i.e. microscopy, a rapid diagnostic test, or a molecular diagnostic test [56].

#### Population

Population estimates for each year were calculated by extrapolating from the 2019 Population and Housing Census [54], using annual population growth rates sourced from the United Nations database [57].

#### Environmental, climatic, and socio-economic data

A Google Earth Engine (GEE) script was developed to extract monthly environmental, climatic, and socio-economic proxy data for a region of interest encompassing Central Vietnam from 2018 to 2022. These data were used for Bayesian spatio-temporal analysis.

The data collection process encompassed multiple facets. Land surface temperature information was acquired from the MODIS MYD11A1 Version 6.1 product, sourced from the Aqua satellite, offering daily LST data at a 1-km resolution within a 1,200 by 1,200 km grid, covering both daytime and nighttime periods. Two-metre air temperature data, crucial for reflecting human-experienced conditions, was drawn from ERA5-Land, part of the ECMWF ERA5 climate reanalysis, providing monthly minimum (TMIN) and maximum (TMAX) air temperature readings that were subsequently converted from Kelvin to degrees Celsius (°C). NDVI was calculated through Landsat 8 Collection 2 Tier 1 imagery, with monthly median values computed in GEE using the NDVI formula. Missing NDVI data resulting from high cloud cover was remedied via linear interpolation. Similarly, NDWI data, responsive to changes in vegetation canopy moisture content, were obtained, and calculated using Near-Infrared (Near-IR) and Infrared (IR) bands. Precipitation (PREC) data was acquired monthly from the Climate Hazards Group InfraRed Precipitation with Station data (CHIRPS), an amalgamation of high-resolution satellite imagery and ground station data spanning over three decades. Furthermore, NTL data, essential for radiance assessments, was collected in the form of monthly composite images using the Visible Infrared Imaging Radiometer Suite (VIIRS) Day/Night Band (DNB), with datasets provided by the Earth Observation Group at the Payne Institute for Public Policy, Colorado School of Mines. Lastly, altitude (ALT) data were sourced from the WorldClim database, offering spatial resolution at 30 arc-seconds, roughly equivalent to 1 km.

#### Raw standardized morbidity ratios

An initial descriptive analysis of malaria incidence was conducted. To calculate the raw standardized morbidity ratios (SMR) for each district, this study applied the following formula:

$$Y_i = \frac{{O_i }}{{E_i }}$$

where *Y* is the overall SMR in district *i*, *O* is the total number of observed malaria cases in the district and *E* is the expected number of malaria cases in the district across the study period. The expected number was calculated by multiplying the average population for each district by the national incidence over the study period [34].

#### Independent variable selection

The independent variables were selected based on Akaike's information criterion (AIC) and Bayesian information criterion (BIC). The dependent variable was the number of malaria cases for each species, while the independent variables encompass environmental factors (NDVI, NDWI, LST daytime/nighttime), climatic variables (TMAX, TMIN, PREC), and a socio-economic variable (NTL). These variables were assessed both without lag and with 1, 2, 3, 4, 5, and 6-month lag times, incorporated into univariate models. Significant variables (p < 0.05) from the univariate models, with the lowest AIC and BIC values, were chosen for the final models. Additionally, Pearson correlation analyses were performed to evaluate collinearity among all the included variables. All preliminary statistical analyses were carried out using STATA software version 18.0 (Stata Corporation, College Station, TX, USA).

#### Exploration of seasonal patterns and temporal trends

The average monthly malaria cases were calculated by *Plasmodium* species from January 2018 to December 2022 and visualized temporal patterns alongside climate data. To analyse malaria incidence trends, this study used seasonal trend decomposition with locally weighted regression, which separated data into seasonal patterns (*S*_*t*_), temporal trends (*T*_*t*_), and residual variability (*R*_*t*_) components. This enabled the acquisition of insights into how malaria cases evolved over time:

$${\text{Y}}_{\text{t}} {\text{ = S}}_{\text{t}} {\text{ + T}}_{\text{t}} {\text{ + R}}_{\text{t}}$$

The seasonal extraction was performed using the "periodic" parameter setting while leaving all other parameters at their default values using RStudio version 4.3.1. The time series data was log transformed for this analysis  [58].

#### Spatio‑temporal model

After accounting for the large number of zero cases (8,486 [87.9%]) in the study, Zero-inflated Poisson (ZIP) regression model was selected over Poisson regression. ZIP models were developed in the Bayesian statistical software WinBUGS version 1.4 (Medical Research Council, Cambridge, UK and Imperial College London, UK) for *P. falciparum* and *P. vivax*, respectively. Alternative models were tested for each species, including models that include environmental, climatic, and socio-economic variables, which were selected in the independent variable selection section as explanatory variables, and spatially structured and unstructured random effects. In total, four models were designed to serve for this analysis. Specifically, Model I incorporated only independent variables as explanatory factors. Model II introduced spatially structured random effects. Model III encompassed both explanatory factors and spatially structured random effects. Model IV was the same as Model III with a combination of a spatiotemporal random effect that estimated spatial variability in district temporal trends. The final model for each species was selected with the lowest Deviance Information Criterion (DIC) value.

The most comprehensive model, aiming to predict observed malaria counts, *Y*, for *i*^*th*^ district (*i* = 1…161) in the *j*^*th*^ month (from January 2018 to December 2022) was structured as follows:

$$\begin{gathered} P(Y_{ij} = y_{ij} ) = \left\{ {\begin{array}{*{20}c} {\omega + 1\left( {1 - \omega } \right)e^{ - \mu } ,y_{ij} = 0} \\ {\left( {1 - \omega } \right)e^{ - \mu } {{\mu _{ij} ^{y_{ij} } } / {y_{ij} ,y_{ij} > 0}}} \\ \end{array} } \right. \hfill \\ Y_{ij} \sim Poisson\left( {\mu _{ij} } \right) \hfill \\ {\text{log }}\left( {\mu _{ij} } \right){\mkern 1mu} = {\text{ log}}\left( {E_{ij} } \right){\mkern 1mu} + \theta _{ij} \hfill \\ \theta _{ij} = \alpha + \beta _1 \times {\text{trend}}_j + \beta _2 \times {\text{ Var}}1_{ij} + {\mkern 1mu} \ldots + \beta _n \times {\text{ Var}}n_{ij} + {\text{ u}}_i + {\text{ v}}_i + {\text{ w}}_{ij} \hfill \\ \end{gathered}$$

where* E* is the expected number of cases (acting as an offset to control for population size) and *θ* is the mean log relative risk (RR); *α* is the intercept, and *β*_*1*_*, β*_*2*_*,…, β*_*n*_ the coefficients for selected covariates; Var1_*ij*_ to Varn_*ij*_ represents co-variates in districts *i* and month *j*; u_*i*_ is the unstructured random effect (assumed to have a mean of zero and variance *σ*_*u*_^*2*^), v_*i*_ is the spatially structured random effect (assumed to have a mean of zero and variance *σ*_*v*_^*2*^) and w_*ij*_ is the spatiotemporal random effect (assumed to have a mean of zero and variance of *σ*_*w*_^*2*^).

A conditional autoregressive (CAR) prior structure was employed to model the spatially structured random effect and the spatiotemporal random effect (allowing for the smoothing of temporal trends at the district level). This approach involves using an adjacency weights matrix to quantify the spatial relationships among the districts. A weight of "1" was assigned when two districts share a border, and "0" when they do not. For the intercept, a flat prior distribution was set, while for the coefficients, a normal prior distribution was specified. In terms of the priors for the precision of the unstructured and spatially structured random effects and the spatiotemporal random effects, non-informative gamma distributions were employed with shape and scale parameters set to 0.01. In the modelling process, models were also developed excluding the structured and unstructured random effects. This was done to assess the  model fitness.

To ensure the stability of the modelling process, a burn-in phase was initiated comprising 10,000 iterations to stabilize the model, discarding the outcomes from this phase. Following this, consecutive blocks of 20,000 iterations were performed, scrutinizing them for convergence. Model convergence was  done through visual examination of posterior density, history plots, and Gelman-Rubin statistics. In this study, convergence was achieved after 100,000 iterations for each model. After convergence (at 100,000 iterations)  posterior distributions of each model parameter were stored and summarized as posterior mean and a 95% credible interval (CrI), posterior maps and trend.

Throughout all analyses, an α-level of 0.05 was adopted to indicate statistical significance (as indicated by 95% CrI for relative risks [RR] that excluded 1). The posterior means of the unstructured and structured random effects, and trends were created with  ArcGIS Pro 3.2 software (ESRI, Redlands, CA).

## Results

### Descriptive analysis

There was a total of 5,985 *Plasmodium falciparum* and 2,623 *Plasmodium vivax* cases during the study period. The ratio of *P. falciparum* to *P. vivax* increased from 2.0 (67.0% *P. falciparum*; 2,011 cases) in 2018 to 4.55 (82.0% *P. falciparum*; 220 cases) in 2022. The incidence rate of *P. falciparum* declined from 1.21 per 10,000 in 2018 to 0.13 per 10,000 in 2022, while the incidence rate of *P. vivax* varied throughout the same time with 0.60 and 0.03 per 10,000 (range 0.03 to 0.66 per 10,000) in 2018 and 2022, respectively (Table 1).

**Table 1:** Malaria incidence during the study period (year 2018—2022)

Year	*Plasmodium falciparum*			*Plasmodium vivax*		
	Case	Proportion by year	Incidence per 10,000	Case	Proportion by year	Incidence per 10,000
2018	2,011	0.67	1.21	996	0.33	0.60
2019	2,823	0.72	1.69	1,100	0.28	0.66
2020	740	0.67	0.44	367	0.33	0.22
2021	191	0.63	0.11	113	0.37	0.07
2022	220	0.82	0.13	47	0.18	0.03
Total	5,985			2,623		

The analysis of this study encompassed a range of environmental, climatic, and socio-economic variables across Central Vietnam from 2018 to 2022 (Supplementary Table 1). ALT in the study area ranged from 5.14 to 1339.56 m above sea level (median: 291.72 m, interquartile range [IQR]: 105.30—526.80 m). LST varied considerably, with daytime temperatures (LSTd) ranging from 18.02 °C to 47.53 °C (median: 30.70 °C) and nighttime temperatures (LSTn) from 5.67 °C to 27.48 °C (median: 20.21 °C). TMAX ranged from 21.77 °C to 39.89 °C (median: 31.38 °C). PREC showed high variability, ranging from 0.06 mm to 298.51 mm per month (median: 21.63 mm, IQR: 7.71—43.48 mm).

For the *P. falciparum* model (Supplementary Table 2), key variables included NDVI without lag (median: 0.02, range: -0.13 to 0.12), NTL with a 3-month lag (median: 0.49 nanowatts/cm^2^/sr, range: -0.04 to 187.65), and TMAX with a 6-month lag (median: 31.44 °C, range: 22.64 °C to 39.89 °C). For the *P. vivax* model (Supplementary Table 3), notable variables included LSTd with a 6-month lag (median: 30.71 °C, range: 10.39 °C to 47.53 °C) and NDVI with a 1-month lag (median: 0.45, range: – 0.01 to 0.78). Both models incorporated altitude and PREC with a 6-month lag as common factors.

### Raw standardized morbidity ratios of malaria

**Fig. 2:**
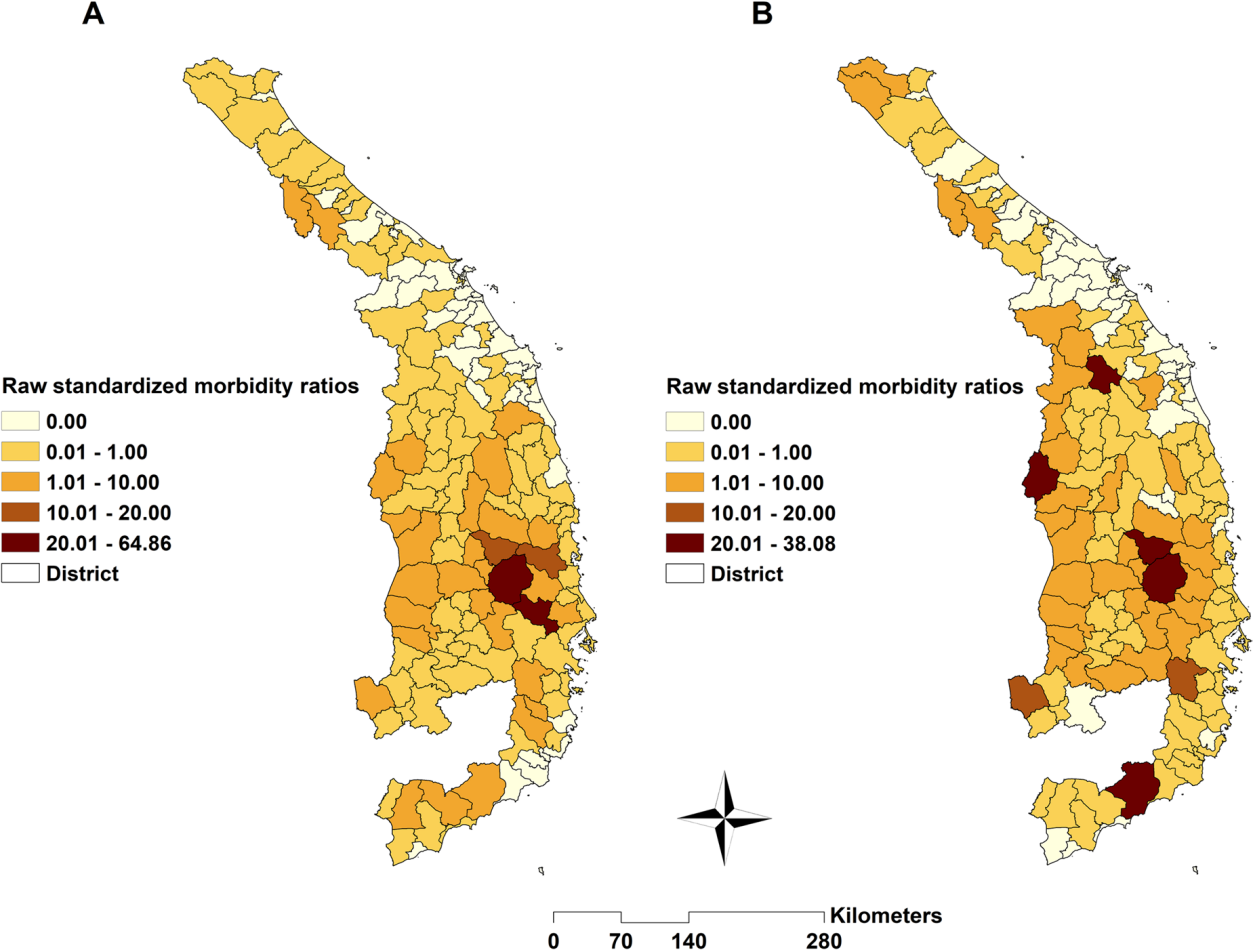
Raw standardized morbidity ratios of **A** *Plasmodium falciparum* and **B** *Plasmodium vivax*, classified by districts in Central Vietnam, 2018–2022

A common trend evident from the spatial distribution map of *P. falciparum*'s SMR indicates high-risk regions in the Southeast, Central Highlands, and South-Central Coast regions, while lower-risk areas are observed in the northern part of the Central region. Similarly, the distribution of *P. vivax* SMR mirrors that of *P. falciparum*, but with an additional expansion of high-risk regions to the Northwest, particularly along the international border shared with Laos and Cambodia (Fig. 2).

### Time‑series decompositions

**Fig. 3:**
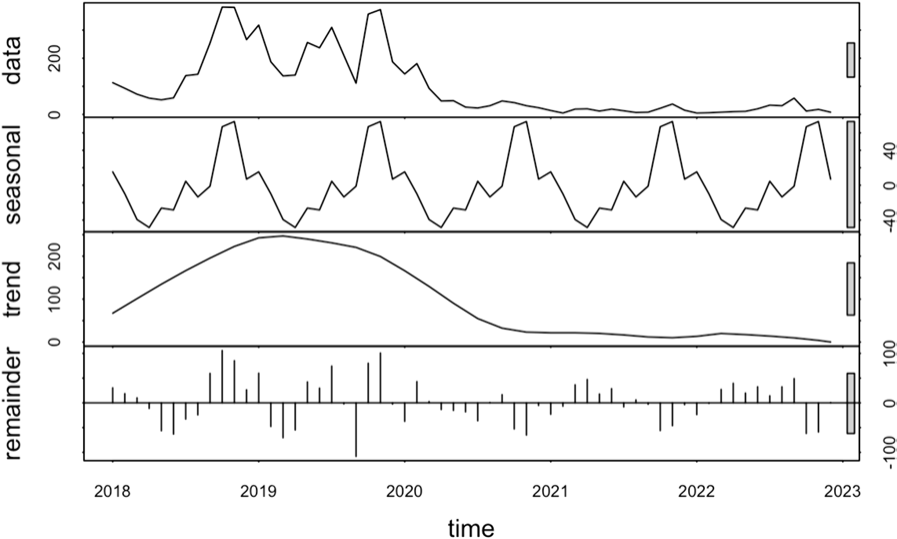
Decomposed monthly *Plasmodium falciparum* incidence in Central Vietnam from 2018 to 2022

**Fig. 4:**
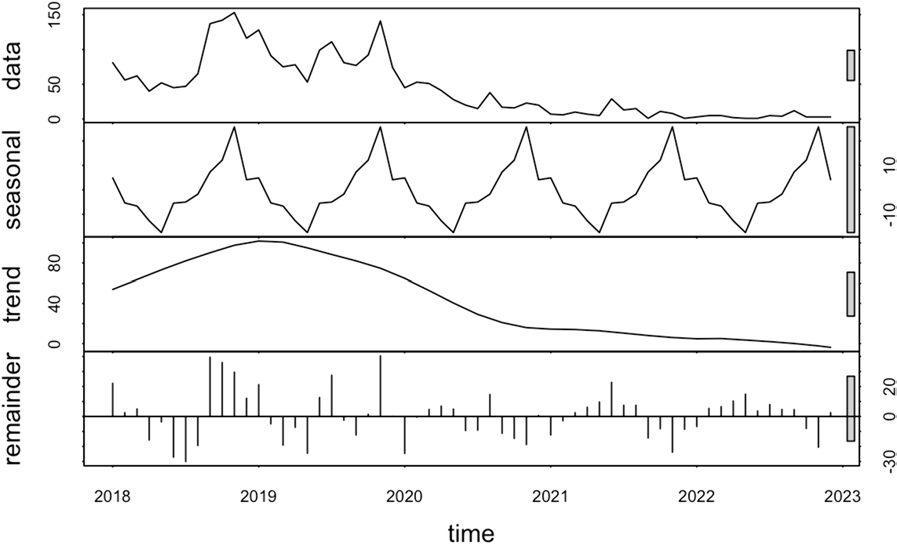
Decomposed monthly *Plasmodium vivax* incidence in Central Vietnam from 2018 to 2022

Time-series decompositions of the raw data during the period of study revealed a conspicuous seasonal pattern for both *P. falciparum* and *P. vivax*. When examining *P. falciparum*, the seasonal pattern reveals up to three distinct peaks: one large peak and two smaller ones before and after this main peak (Fig. 3). In contrast, when observing *P. vivax*, the data shows just two peaks, a significant one and a smaller counterpart (Fig. 4). It's worth noting that both species exhibit their primary seasonal peaks during the final months of the year. The inter-annual pattern showed malaria incidence gradually increased and peaked in 2019 then gradually decreased in the following years for both *P. falciparum* and *P. vivax* species (Figs. 3 and 4).

### Spatio‑temporal model

Among the four models assessed for spatio-temporal modelling, Model IV, incorporating the unstructured and the spatially structured random effect, and the spatio-temporal random effect had the best-fit model for both *P. falciparum* and *P. vivax*, because of having the lowest DIC. *Plasmodium falciparum* risk increased by 5.42-fold (95% CrI 4.37, 6.74) for each 1-unit increase of NDVI without lag. There was a protective effect with a decrease in risk of 1% (95% CrI 0%, 1%) for a 1 mm increase in PREC with a 6-month lag. Risk increased by 8% (95% CrI 7%, 9%) for every 1ºC increase in TMAX at a 6-month lag. There was no statistically significant association between the risk of *P. falciparum* infection and NTL (Table 2).

**Table 2:** Parameters estimate from Bayesian Zero-Inflated Poisson regression models of *Plasmodium falciparum* cases reported by month and district, Central Vietnam, 2018—2022

Variables	Model I RR (95%CrI)	Model II RR (95%CrI)	Model III RR (95%CrI)	Model IV RR (95%CrI)
Intercept (α)*	−1.19 (−1.48, 0.92)	−1.29 (−1.49, 1.10)	−1.31 (−1.60, 1.04)	−2.07 (−2.42, 1.73)
Mean monthly trend	0.52 (0.50, 0.53)	0.51 (0.47, 0.56)	0.52 (0.48, 0.55)	0.26 (0.22, 0.30)
ALT (masl)	1.00 (0.15, 6.37)	1.00 (0.15, 6.45)	1.00 (0.15, 6.51)	1.01 (0.16, 6.46)
NDVI (unit) (without lag)	4.69 (4.01, 5.49)	4.76 (3.66,6.22)	4.72 (3.84, 5.85)	5.42 (4.37, 6.74)
NTL (nanowatts/cm^2^ /sr) (3-month lag)	1.00 (0.99, 1.00)	1.00 (0.99, 1.00)	1.00 (0.99, 1.00)	1.00 (0.99, 1.00)
PREC (mm) (6-month lag)	0.99 (0.99, 1.00)	0.99 (0.99, 1.00)	0.99 (0.99, 1.00)	0.99 (0.99, 1.00)
TMAX (ºC) (6-month lag)	1.07 (1.06, 1.08)	1.07 (1.06, 1.09)	1.07 (1.06, 1.09)	1.08 (1.07, 1.09)
Probability of extra zero	1.58 (1.54, 1.62)	1.58 (1.51, 1.65)	1.58 (1.52, 1.63)	1.45 (1.40, 1.50)
Heterogeneity*				
Unstructured	0.24 (0.18, 0.30)		0.55 (0.29, 1.11)	0.44 (0.27, 0.71)
Structured		0.08 (0.06, 0.10)	0.34 (0.11, 0.77)	0.33 (0.12, 0.72)
Structured (trend)				1.44 (0.90, 2.13)
DIC	11,660.4	11,771.5	11,696	11,259.8

^*^Co-efficient, RR—Relative Risk, CrI—Credible Interval, DIC—Deviation Information Criteria, ALT – Altitude, NDVI—Normalized difference vegetation index, NTL – Nighttime lights, PREC – Precipitation, TMAX – Maximum air temperature

For *P. vivax*, a 1-unit increase in NDVI at a 1-month lag was significantly associated with a 3.79-fold increase (95% CrI 2.95, 4.90) in risk of *P. vivax*. In addition, the risk increased by 6% (95% CrI 5%, 7%) and 3% (95% CrI 1%, 5%) for each 1ºC increase in LSTd with a 6-month lag and TMAX at a 4-month lag, respectively. No statistically significant association between the risk of *P. vivax* infection and PREC with a 6-month lag (Table 3).

**Table 3:** Parameters estimate from Bayesian Zero-inflated Poisson regression models of *Plasmodium vivax* cases reported by month and district, Central Vietnam, 2018—2022

Variables	Model I RR (95%CrI)	Model II RR (95%CrI)	Model III RR (95%CrI)	Model IV RR (95%CrI)
Intercept (α)*	– 0.94 (– 1.25, – 0.64)	– 1.04 (– 1.26, – 0.83)	– 0.96 (– 1.27, – 0.64)	– 1.26 (– 1.59, – 0.93)
Mean monthly trend	0.42 (0.40, 0.44)	0.42 (0.37, 0.47)	0.42 (0.39, 0.44)	0.34 (0.30, 0.39)
ALT (masl)	0.99 (0.16, 6.30)	1.00 (0.16, 6.43)	1.00 (0.15, 6.53)	1.01 (0.16, 6.46)
NDVI (Unit) (1-month lag)	3.34 (2.66, 4.21)	3.41 (2.54, 4.63)	3.36 (2.66, 4.25)	3.79 (2.95, 4.90)
LSTd (ºC) (6-month lag)	1.05 (1.04, 1.06)	1.05 (1.04, 1.06)	1.05 (1.04, 1.06)	1.06 (1.05, 1.07)
PREC (mm) (6-month lag)	1.00 (1.00, 1.00)	1.00 (1.00, 1.00)	1.00 (1.00, 1.00)	1.00 (1.00, 1.00)
TMAX (ºC) (4-month lag)	1.04 (1.02, 1.05)	1.04 (1.02, 1.06)	1.04 (1.02, 1.05)	1.03 (1.01, 1.05)
Probability of extra zero	1.43 (1.39, 1.48)	1.43 (1.36, 1.51)	1.43 (1.38, 1.48)	1.42 (1.37, 1.47)
Heterogeneity*				
Unstructured	0.29 (0.22, 0.37)		0.38 (0.24, 0.66)	0.37 (0.23, 0.66)
Structured		0.08 (0.06, 0.11)	302.60 (0.22, 1,600)	23.12 (0.17, 136.80)
Structured (trend)				0.91 (0.60, 1.33)
DIC	7540.7	7585.2	7543.7	7389.2

*Co-efficient,* RR* Relative Risk,* CrI* Credible Interval,* DIC* Deviation Information Criteria,* ALT* Altitude,* NDVI* Normalized difference vegetation index,* LSTd* Land surface temperature at daytime,* PREC* Precipitation,* TMAX* Maximum air temperature

**Fig. 5:**
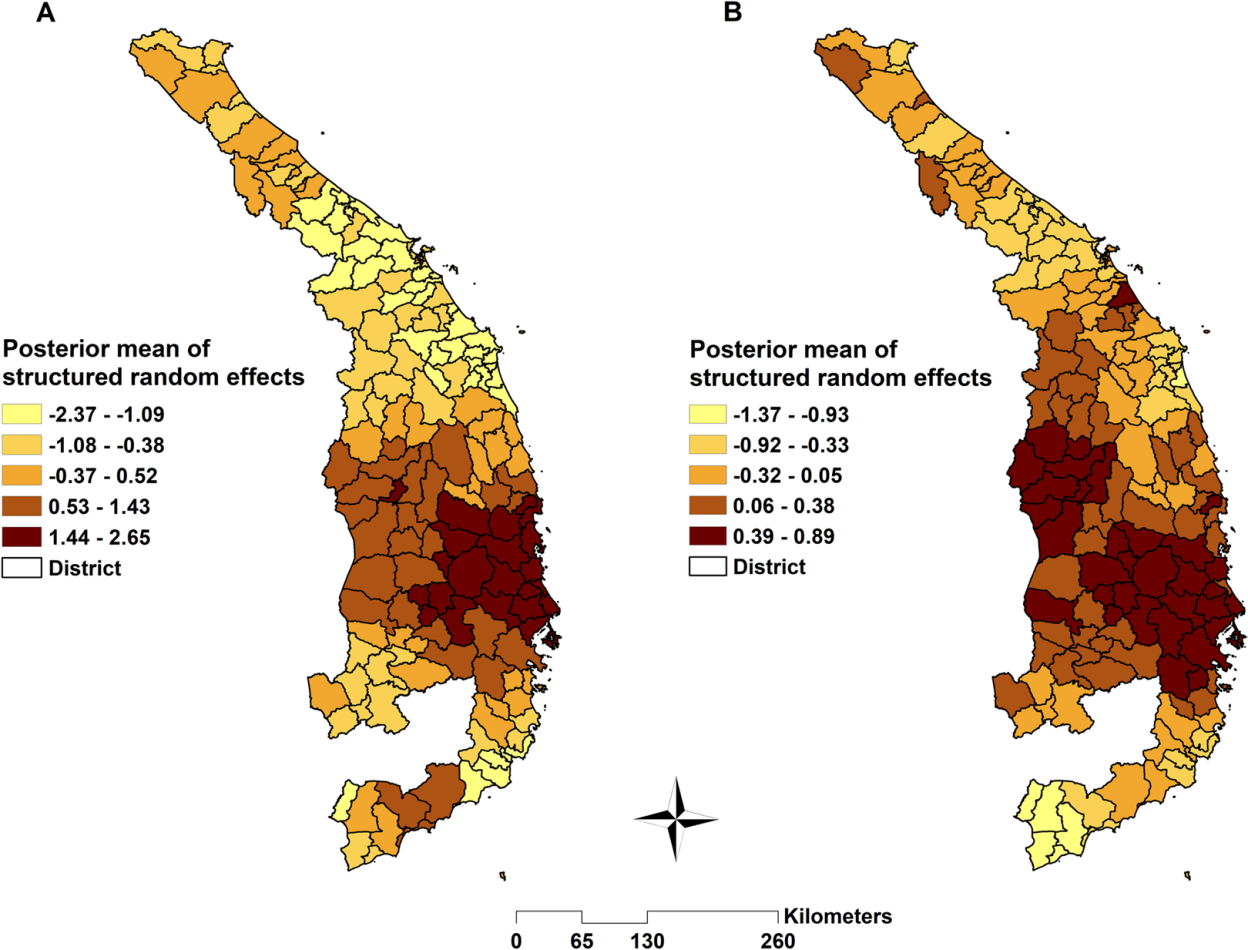
Spatial distribution of the posterior means of structured random effects for (**A**) *Plasmodium falciparum* and (**B**) *Plasmodium vivax*

Estimation of the spatially auto-correlated random effect (v*i*) showed a higher mean malaria risk of both species in the Central Highland and southeast parts of Central Vietnam and a lower risk in the northern and north-western areas (Fig. 5).

**Fig. 6:**
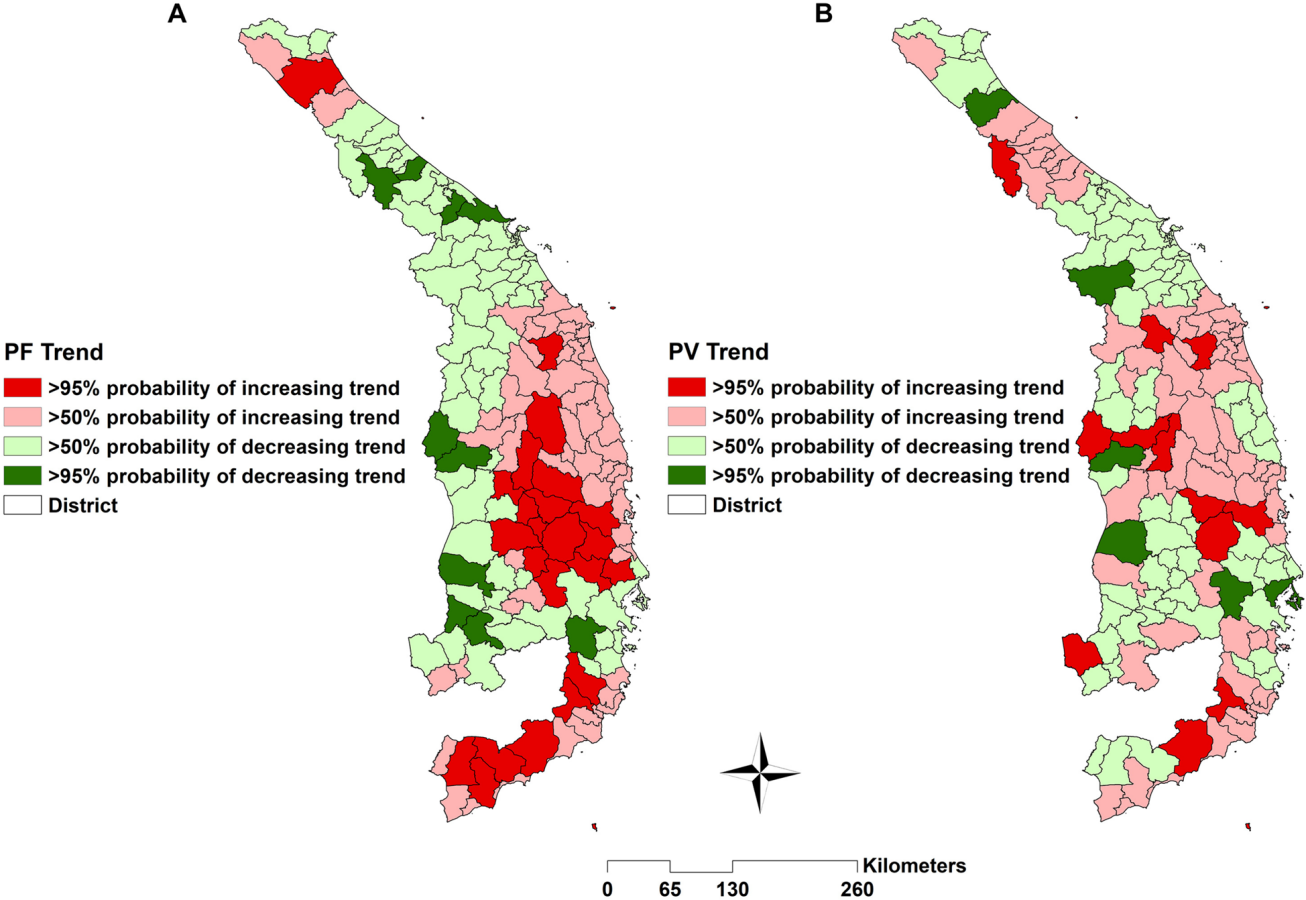
Trend analysis of (**A**) *Plasmodium falciparum* and (**B**) *Plasmodium vivax* in Central Vietnam, from 2018 to 2022

According to trend analysis, Model IV showed a significant, positive temporal trend in counts of cases of both types of malaria over the study period. For *P. falciparum,* there was > 95% probability of a higher than the regional average trend of *P. falciparum* in 26/161 districts and 57/161 districts have > 50% probability of a higher than the regional average trend, mostly located in the east-central part and the southern part of Central region. While there were 10/161 and 68/161 districts have > 95% and > 50% probability of a lower than the regional average trend, respectively, distributed mostly in the north, the west and southwestern parts of Central Vietnam. In contrast, districts had increasing and decreasing probability trends comparable to regional trends of *P. vivax* scattered throughout the region. There were 12/161 districts have > 95% probability of a higher than the regional average trend, whereas 6/161 districts have > 95% probability of a lower than the regional average trend. (Fig. 6).

## Discussion

The findings of this study provide valuable insights into the spatial and temporal dynamics of *P. falciparum* and *P. vivax* malaria in Central Vietnam from 2018 to 2022. This analysis showed reduction in overall cases and annual seasonal peaks in malaria incidence and spatial variations in distribution. There was a significant association between malaria infection with TMAX, NDVI, PREC, and LST during the daytime.

Despite an overall decline in malaria cases, a notable shift in the ratio of *P. falciparum* to *P. vivax* was observed, increasing from 2.0 in 2018 to 4.55 in 2022. This trend suggests a disproportionate reduction in *P. vivax* cases compared to *P. falciparum*. One of the possible reasons is *P. falciparum* is more sensitive to climatic conditions compared to *P. vivax* as reported by some other studies [59–62]. A plausible reason could be, *P. vivax* is known for relapse when the dormant (in hypnozoite form) is reactivated. In the dormant state, infection can hide in the liver, lying and protected from the external environment. However, *P. vivax* infection was positively associated with climatic and environmental factors in this study. Other factors might be related to drug resistance, dihydroartemisinin-piperaquine (DHA-PPQ), a malaria treatment, is becoming less effective in the eastern Greater Mekong Subregion, particularly in Thailand, Cambodia, and Vietnam [63]. This ineffectiveness is related to increased treatment failures. Van der Pluijm et al*.* [63] identified the rise of specific genetic markers associated with resistance to artemisinin and piperaquine, indicating growing drug resistance in the region. Treatment failures with DHA-PPQ are linked to the presence of plasmepsin-2/3 amplifications and specific mutations in the *crt* gene. This suggests that a highly drug-resistant lineage of the malaria parasite *P. falciparum* is emerging in the region and developing new resistance mechanisms [63].

The results of this study showed that an increase in the NDVI was significantly associated with the increase in malaria risk in both *P. falciparum* and *P. vivax*. Several potential mechanisms could explain this association. Firstly, dense vegetation can provide breeding sites and resting places for *Anopheles* mosquitoes. Higher NDVI values may indicate more abundant vegetation, which could lead to increased mosquito populations and, consequently, a higher risk of malaria transmission. Vegetation can influence local microclimates, potentially creating conditions more favorable for mosquito survival and reproduction [64]. Dense vegetation can retain moisture and shade, creating cooler and more humid environments that are preferred by *Anopheles* mosquitoes [65]. Changes in vegetation cover, often associated with deforestation or agricultural expansion, can alter human behaviour and land use patterns, potentially increasing exposure to mosquito bites. Deforestation may lead to increased human contact with mosquitoes in previously undisturbed areas [66]. Dense vegetation can also influence the abundance and distribution of other organisms, including predators and competitors of *Anopheles* mosquitoes [67]. Changes in these ecological interactions could indirectly affect mosquito populations and malaria transmission risk.

A one cm increase in PREC with a 6-month lag significantly reduced the risk of *P. falciparum* infection by 10%, while no significant association was observed for *P. vivax* infection with the same PREC lag. The potential protective effect of increased PREC against *P. falciparum* malaria risk can be attributed to several mechanisms [68, 69]. Enhanced PREC may dilute mosquito breeding sites, rendering them less suitable for malaria parasite development, consequently reducing the number of infective mosquitoes and lowering the transmission risk [70]. Heavy PREC can flush out mosquito larvae and pupae from breeding areas, effectively reducing mosquito populations [16, 71]. This reduced mosquito density correlates with a decreased risk of malaria transmission. Moreover, increased precipitation can impact vegetation growth and distribution, potentially creating less favourable conditions for mosquito survival [72]. Changes in PREC patterns might also influence human behaviour, potentially reducing exposure to mosquito bites, as people may avoid outdoor activities during heavy rains [70, 73]. Meanwhile, *P. vivax* has a complex life cycle that includes a dormant liver stage, which could make it less susceptible to environmental fluctuations than *P. falciparum*. This difference in parasite biology may contribute to the observed disparity in the association with PREC. In addition, the epidemiology of *P. vivax* infection may be more influenced by factors other than PREC, such as human movement and population immunity.

This study also showed that an increase in monthly TMAX was significantly associated with an increase in risk for both *P. falciparum* and *P. vivax*. It is noted that higher temperatures will increase the number of cases of malaria infections because malaria parasites will develop faster in the mosquitoes and the ability of mosquitoes to acquire malaria parasites will be increased by increased blood-sucking activities [74]. However, the latest studies showed that temperature has a more complicated effect on malaria transmission. As temperature rises, parasites develop faster, but fewer of them become infectious. Therefore, the influence of an increase in temperature on malaria dynamics and distribution remains controversial. Wang et al*.* showed the diverse effects of rising temperatures on malaria epidemic trends in tropical regions with great heterogenicity in altitude [75].

LST during the daytime has a positive correlation with *P. vivax* infection risk. This study showed that the *P. vivax* risk increased by 6% (95% CrI 5%, 7%) for each 1ºC increase in the LSTd with a 6-month lag. Previous studies also showed that higher LST can increase the risk of malaria transmission [7], especially in areas with low vegetation cover and moisture after the rainy season [21].

This study’s analysis offers significant improvements over the study by Wangdi et al*.* [34] by incorporating climatic, environmental, and socio-economic variables with extended lag periods. This approach enhances the model's capacity to account for the complex and delayed effects of these factors on malaria transmission, potentially leading to more accurate predictions and better-informed public health decisions. A novel aspect of this study is the inclusion of nighttime light intensity as an indicator of socio-economic development and urbanization. This addition allows for a more multidimensional analysis, considering not only environmental and climatic factors but also socio-economic conditions. However, the results of this study indicate that the association between NTL and malaria infection risk is not statistically significant. This suggests that while socio-economic development is an important factor in public health, it may not have a direct or immediate impact on malaria transmission in the study area.

While this study provides valuable insights into the environmental, climatic, and socio-economic factors affecting malaria transmission several limitations remain. Firstly, it does not explicitly consider the impact of vector control measures, such as insecticide-treated bed nets and indoor residual spraying, which can significantly influence malaria transmission. Additionally, population estimates were extrapolated from census data, potentially introducing uncertainty due to demographic shifts. The accuracy and completeness of malaria case records, a cornerstone of our analysis, can vary and may not capture the full extent of cases. Furthermore, the study did not account for the indirect consequences of the COVID-19 pandemic on healthcare systems and malaria control efforts during 2020–2021. Moreover, the role of drug resistance to DHA-PPQ in Vietnam was not accounted in the model and is recommended to be explored in future studies. Acknowledging these limitations underscores the need for more comprehensive data, improved modelling approaches, and ongoing research to enhance the understanding of malaria dynamics and inform more effective control and elimination strategies in Central Vietnam.

## Conclusion

The findings of this study will not only contribute to a deeper understanding of the determinants of malaria transmission in Central Vietnam but will also provide actionable insights for public health officials, policymakers, and healthcare practitioners. These insights can guide resource allocation, the development of targeted interventions, and the design of adaptive strategies to effectively eliminate malaria in this region.

### Supplementary Information

**Additional file 1.**

## Data Availability

The original contributions presented in the study are included in the article. Further inquiries can be directed to the first author and corresponding author.

## References

[1] WHO. World malaria report 2022. Geneva, World Health Organization, 2022.

[2] Erhart A, Ngo DT, Phan VK, Ta TT, Van Overmeir C, Speybroeck N, et al. Epidemiology of forest malaria in central Vietnam: a large scale cross-sectional survey. Malar J. 2005;4:58. 16336671 10.1186/1475-2875-4-58 PMC1325238

[3] Sanh NH, Van Dung N, Thanh NX, Trung TN, Van Co T, Cooper RD. Forest malaria in central Vietnam. Am J Trop Med Hyg. 2008;79:652–4. 18981498 10.4269/ajtmh.2008.79.652

[4] le Hung Q, Vries PJ, Giao PT, Nam NV, Binh TQ, Chong MT, et al. Control of malaria: a successful experience from Viet Nam. Bull World Health Organ. 2002;80:660–6. 12219158 PMC2567582

[5] Bui HM, Clements AC, Nguyen QT, Nguyen MH, Le XH, Hay SI, et al. Social and environmental determinants of malaria in space and time in Viet Nam. Int J Parasitol. 2011;41:109–16. 20833173 10.1016/j.ijpara.2010.08.005 PMC3086784

[6] Do Manh C, Beebe NW, Van Thi VN, Le Quang T, Lein CT, Van Nguyen D, et al. Vectors and malaria transmission in deforested, rural communities in north-central Vietnam. Malar J. 2010;9:259. 20846447 10.1186/1475-2875-9-259 PMC2945362

[7] McMahon A, Mihretie A, Ahmed AA, Lake M, Awoke W, Wimberly MC. Remote sensing of environmental risk factors for malaria in different geographic contexts. Int J Health Geogr. 2021;20:28. 34120599 10.1186/s12942-021-00282-0 PMC8201719

[8] Liu Z, Wang S, Zhang Y, Xiang J, Tong MX, Gao Q, et al. Effect of temperature and its interactions with relative humidity and rainfall on malaria in a temperate city Suzhou. China Environ Sci Pollut Res Int. 2021;28:16830–42. 33394450 10.1007/s11356-020-12138-4

[9] Okuneye K, Gumel AB. Analysis of a temperature- and rainfall-dependent model for malaria transmission dynamics. Math Biosci. 2017;287:72–92. 27107977 10.1016/j.mbs.2016.03.013

[10] Liu Q, Wang Y, Deng J, Yan W, Qin C, Du M, et al. Association of temperature and precipitation with malaria incidence in 57 countries and territories from 2000 to 2019: a worldwide observational study. J Glob Health. 2024;14:04021. 38385445 10.7189/jogh.14.04021 PMC10882640

[11] Kabaria CW, Gilbert M, Noor AM, Snow RW, Linard C. The impact of urbanization and population density on childhood *Plasmodium falciparum* parasite prevalence rates in Africa. Malar J. 2017;16:49. 28125996 10.1186/s12936-017-1694-2 PMC5270336

[12] Zhao X, Thanapongtharm W, Lawawirojwong S, Wei C, Tang Y, Zhou Y, et al. Malaria risk map using spatial multi-criteria decision analysis along Yunnan Border during the pre-elimination period. Am J Trop Med Hyg. 2020;103:793–809. 32602435 10.4269/ajtmh.19-0854 PMC7410425

[13] Mordecai EA, Paaijmans KP, Johnson LR, Balzer C, Ben-Horin T, de Moor E, et al. Optimal temperature for malaria transmission is dramatically lower than previously predicted. Ecol Lett. 2013;16:22–30. 23050931 10.1111/ele.12015

[14] Lafferty KD. The ecology of climate change and infectious diseases. Ecology. 2009;90:888–900. 19449681 10.1890/08-0079.1

[15] Shapiro LLM, Whitehead SA, Thomas MB. Quantifying the effects of temperature on mosquito and parasite traits that determine the transmission potential of human malaria. PLoS Biol. 2017;15: e2003489. 29036170 10.1371/journal.pbio.2003489 PMC5658182

[16] Lemma W. Description of malaria epidemics and normal transmissions using rainfall variability in Gondar Zuria highland District. Ethiopia Heliyon. 2021;7: e07653. 34409176 10.1016/j.heliyon.2021.e07653 PMC8361060

[17] Liu J, Chen XP. Relationship of remote sensing normalized differential vegetation index to *Anopheles* density and malaria incidence rate. Biomed Environ Sci. 2006;19:130–2. 16827184

[18] Okiring J, Routledge I, Epstein A, Namuganga JF, Kamya EV, Obeng-Amoako GO, et al. Associations between environmental covariates and temporal changes in malaria incidence in high transmission settings of Uganda: a distributed lag nonlinear analysis. BMC Public Health. 2021;21:1962. 34717583 10.1186/s12889-021-11949-5 PMC8557030

[19] Wayant NM, Maldonado D, de Arias A, Cousiño B, Goodin DG. Correlation between normalized difference vegetation index and malaria in a subtropical rain forest undergoing rapid anthropogenic alteration. Geospat Health. 2010;4:179–90. 20503187 10.4081/gh.2010.199

[20] Merkord CL, Davis JK, Wimberly MC. Evaluation of environmentally driven models for early warning of malaria: an exploratory study. Lancet. 2017;389:S13. 10.1016/S0140-6736(17)31125-X

[21] Garske T, Ferguson NM, Ghani AC. Estimating air temperature and its influence on malaria transmission across Africa. PLoS ONE. 2013;8: e56487. 23437143 10.1371/journal.pone.0056487 PMC3577915

[22] Zhao M, Cheng W, Zhou C, Li M, Huang K, Wang N. Assessing spatiotemporal characteristics of urbanization dynamics in Southeast Asia using time series of DMSP/OLS nighttime light data. Remote Sensing. 2018;10:47. 10.3390/rs10010047

[23] Barghini A, de Medeiros BA. Artificial lighting as a vector attractant and cause of disease diffusion. Environ Health Perspect. 2010;118:1503–6. 20675268 10.1289/ehp.1002115 PMC2974685

[24] Reiter P. Climate change and mosquito-borne disease: knowing the horse before hitching the cart. Rev Sci Tech. 2008;27:383–98. 18819667 10.20506/rst.27.2.1804

[25] Caminade C, McIntyre KM, Jones AE. Impact of recent and future climate change on vector-borne diseases. Ann N Y Acad Sci. 2019;1436:157–73. 30120891 10.1111/nyas.13950 PMC6378404

[26] Teklehaimanot HD, Lipsitch M, Teklehaimanot A, Schwartz J. Weather-based prediction of Plasmodium falciparum malaria in epidemic-prone regions of Ethiopia I. Patterns of lagged weather effects reflect biological mechanisms. Malar J. 2004;3:41. 15541174 10.1186/1475-2875-3-41 PMC535540

[27] Bi P, Tong S, Donald K, Parton KA, Ni J. Climatic variables and transmission of malaria: a 12-year data analysis in Shuchen County. China Public Health Rep. 2003;118:65–71. 12604766 10.1016/S0033-3549(04)50218-2 PMC1497511

[28] Paaijmans KP, Blanford S, Chan BH, Thomas MB. Warmer temperatures reduce the vectorial capacity of malaria mosquitoes. Biol Lett. 2012;8:465–8. 22188673 10.1098/rsbl.2011.1075 PMC3367745

[29] Pascual M, Ahumada JA, Chaves LF, Rodo X, Bouma M. Malaria resurgence in the East African highlands: temperature trends revisited. Proc Natl Acad Sci USA. 2006;103:5829–34. 16571662 10.1073/pnas.0508929103 PMC1416896

[30] Beck-Johnson LM, Nelson WA, Paaijmans KP, Read AF, Thomas MB, Bjornstad ON. The effect of temperature on *Anopheles* mosquito population dynamics and the potential for malaria transmission. PLoS ONE. 2013;8: e79276. 24244467 10.1371/journal.pone.0079276 PMC3828393

[31] Midekisa A, Senay G, Henebry GM, Semuniguse P, Wimberly MC. Remote sensing-based time series models for malaria early warning in the highlands of Ethiopia. Malar J. 2012;11:165. 22583705 10.1186/1475-2875-11-165 PMC3493314

[32] Qi Q, Guerra CA, Moyes CL, Elyazar IR, Gething PW, Hay SI, et al. The effects of urbanization on global *Plasmodium vivax* malaria transmission. Malar J. 2012;11:403. 23217010 10.1186/1475-2875-11-403 PMC3528462

[33] Georganos S, Brousse O, Dujardin S, Linard C, Casey D, Milliones M, et al. Modelling and mapping the intra-urban spatial distribution of *Plasmodium falciparum* parasite rate using very-high-resolution satellite derived indicators. Int J Health Geogr. 2020;19:38. 32958055 10.1186/s12942-020-00232-2 PMC7504835

[34] Wangdi K, Canavati SE, Ngo TD, Tran LK, Nguyen TM, Tran DT, et al. Analysis of clinical malaria disease patterns and trends in Vietnam 2009–2015. Malar J. 2018;17:332. 30223843 10.1186/s12936-018-2478-z PMC6142383

[35] Blangiardo M, Cameletti M, Baio G, Rue H. Spatial and spatio-temporal models with R-INLA. Spat Spatiotemporal Epidemiol. 2013;7:39–55. 24377114 10.1016/j.sste.2013.07.003

[36] Ma S, Yu K, Tang ML, Pan J, Hardle WK, Tian M. A Bayesian multistage spatio-temporally dependent model for spatial clustering and variable selection. Stat Med. 2023;42:4794–823. 37652405 10.1002/sim.9889

[37] Greenland S. Bayesian perspectives for epidemiological research: I. Foundations and basic methods. Int J Epidemiol. 2006;35:765–75. 10.1093/ije/dyi312 16446352

[38] Lawson AB. Bayesian disease mapping: hierarchical modeling in spatial epidemiology. 3rd ed. New York: Chapman and Hall/CRC; 2018.

[39] Robert P, Haining GL. Modelling spatial and spatial-temporal data: a Bayesian approach. New York: Chapman and Hall/CRC; 2020.

[40] Gelman A, Carlin JB, Stern HS, Dunson DB, Vehtari A, Rubin DB. Bayesian data analysis. New York: Chapman and Hall/CRC; 2013.

[41] Maude RJ, Ngo TD, Tran DT, Nguyen BTH, Dang DV, Tran LK, et al. Risk factors for malaria in high incidence areas of Viet Nam: a case-control study. Malar J. 2021;20:373. 34535140 10.1186/s12936-021-03908-7 PMC8446736

[42] Kattenberg JH, Erhart A, Truong MH, Rovira-Vallbona E, Vu KAD, Nguyen THN, et al. Characterization of *Plasmodium falciparum* and *Plasmodium vivax* recent exposure in an area of significantly decreased transmission intensity in Central Vietnam. Malar J. 2018;17:180. 29703200 10.1186/s12936-018-2326-1 PMC5923009

[43] Bannister-Tyrrell M, Xa NX, Kattenberg JH, Van Van N, Dung VKA, Hieu TM, et al. Micro-epidemiology of malaria in an elimination setting in Central Vietnam. Malar J. 2018;17:119. 29554901 10.1186/s12936-018-2262-0 PMC5859719

[44] Pilarczyk KW, Nuoi NS. Experience and practices on flood control in Vietnam. Water Int. 2005;30:114–22. 10.1080/02508060508691843

[45] Trinh-Tuan L, Matsumoto J, Ngo-Duc T, Nodzu MI, Inoue T. Evaluation of satellite precipitation products over Central Vietnam. Progr Earth Planet Sci. 2019;6:54. 10.1186/s40645-019-0297-7

[46] Nguyen VT, Dan MN, Doan QV, Tuan BM, Khiem MV, Van KD, Tran TT, et al. Orographic effect and the opposite trend of rainfall in Central Vietnam. Adv Meteorol. 2023;2023:1–12. 10.1155/2023/7256634

[47] Feng Y, Tuan TD, Shi J, Li Z, Maimaitiming M, Jin Y, et al. Progress towards health equity in Vietnam: evidence from nationwide official health statistics, 2010–2020. BMJ Glob Health. 2024;9: e014739. 38503427 10.1136/bmjgh-2023-014739 PMC10952956

[48] Quan NK, Taylor-Robinson AW. Vietnam’s evolving healthcare system: notable successes and significant challenges. Cureus. 2023;15: e40414. 37456482 10.7759/cureus.40414 PMC10348075

[49] Morel CM, Thang ND, Xa NX, le Hung X, le Thuan K, Van Ky P, et al. The economic burden of malaria on the household in south-central Vietnam. Malar J. 2008;7:166. 18752675 10.1186/1475-2875-7-166 PMC2546429

[50] Morrow M, Nguyen QA, Caruana S, Biggs BA, Doan NH, Nong TT. Pathways to malaria persistence in remote central Vietnam: a mixed-method study of health care and the community. BMC Public Health. 2009;9:85. 19309519 10.1186/1471-2458-9-85 PMC2666724

[51] Kar NP, Kumar A, Singh OP, Carlton JM, Nanda N. A review of malaria transmission dynamics in forest ecosystems. Parasit Vectors. 2014;7:265. 24912923 10.1186/1756-3305-7-265 PMC4057614

[52] Thanh PV, Van Hong N, Van Van N, Van Malderen C, Obsomer V, Rosanas-Urgell A, et al. Epidemiology of forest malaria in Central Vietnam: the hidden parasite reservoir. Malar J. 2015;14:86. 25880664 10.1186/s12936-015-0601-y PMC4342195

[53] Brooker S, Hay SI, Bundy DA. Tools from ecology: useful for evaluating infection risk models? Trends Parasitol. 2002;18:70–4. 11832297 10.1016/S1471-4922(01)02223-1 PMC3166848

[54] Vietnam GSOo. Completed Results of the 2019 Viet Nam Population and Housing Census: Statistical Publishing House (Vietnam); 2019.

[55] QGIS.org. QGIS Geographic Information System. QGIS Association; 2023.

[56] WHO. Malaria terminology. 2021 update edn. Geneva: World Health Organization; 2021.

[57] United Nations DoEaSA, Population Division. World Population Prospects 2022: Data Sources. (UN DESA/POP/2022/DC/NO. 9). 2022.

[58] Childs DZ, Cattadori IM, Suwonkerd W, Prajakwong S, Boots M. Spatiotemporal patterns of malaria incidence in northern Thailand. Trans R Soc Trop Med Hyg. 2006;100:623–31. 16406037 10.1016/j.trstmh.2005.09.011

[59] Bi Y, Yu W, Hu W, Lin H, Guo Y, Zhou XN, et al. Impact of climate variability on *Plasmodium vivax* and *Plasmodium falciparum* malaria in Yunnan Province. China Parasit Vectors. 2013;6:357. 24341555 10.1186/1756-3305-6-357 PMC3898806

[60] Galinski MR, Meyer EV, Barnwell JW. *Plasmodium vivax*: modern strategies to study a persistent parasite’s life cycle. Adv Parasitol. 2013;81:1–26. 23384620 10.1016/B978-0-12-407826-0.00001-1

[61] Ohm JR, Baldini F, Barreaux P, Lefevre T, Lynch PA, Suh E, et al. Rethinking the extrinsic incubation period of malaria parasites. Parasit Vectors. 2018;11:178. 29530073 10.1186/s13071-018-2761-4 PMC5848458

[62] Wangdi K, Xu Z, Suwannatrai AT, Kurscheid J, Lal A, Namgay R, et al. A spatio-temporal analysis to identify the drivers of malaria transmission in Bhutan. Sci Rep. 2020;10:7060. 32341415 10.1038/s41598-020-63896-7 PMC7184595

[63] van der Pluijm RW, Imwong M, Chau NH, Hoa NT, Thuy-Nhien NT, Thanh NV, et al. Determinants of dihydroartemisinin-piperaquine treatment failure in *Plasmodium falciparum* malaria in Cambodia, Thailand, and Vietnam: a prospective clinical, pharmacological, and genetic study. Lancet Infect Dis. 2019;19:952–61. 31345710 10.1016/S1473-3099(19)30391-3 PMC6715822

[64] Recopuerto-Medina LM, Gutierrez FCU, San Diego JAS, Alviar NAE, Santos JRM, Dagamac NHA. MaxEnt modeling of the potential risk of schistosomiasis in the Philippines using bioclimatic factors. Parasitol Int. 2024;98: 102827. 38030120 10.1016/j.parint.2023.102827

[65] Obsomer V, Defourny P, Coosemans M. The *Anopheles dirus* complex: spatial distribution and environmental drivers. Malar J. 2007;6:26. 17341297 10.1186/1475-2875-6-26 PMC1838916

[66] Hawkes FM, Manin BO, Cooper A, Daim S, Jelip J, et al. Vector compositions change across forested to deforested ecotones in emerging areas of zoonotic malaria transmission in Malaysia. Sci Rep. 2019;9:13312. 31527622 10.1038/s41598-019-49842-2 PMC6746737

[67] Yasuoka J. Community-based ecosystem management for malaria vector control following deforestation and agricultural development. DSc Thesis: Boston: Harvard School of Public Health; 2005.

[68] Sena L, Deressa W, Ali A. Correlation of climate variability and malaria: a retrospective comparative study Southwest Ethiopia. Ethiop J Health Sci. 2015;25:129–38. 26124620 10.4314/ejhs.v25i2.5 PMC4478264

[69] Wiwanitkit V. Correlation between rainfall and the prevalence of malaria in Thailand. J Infect. 2006;52:227–30. 16442631 10.1016/j.jinf.2005.02.023

[70] Wu Y, Qiao Z, Wang N, Yu H, Feng Z, Li X, et al. Describing interaction effect between lagged rainfalls on malaria: an epidemiological study in south–west China. Malar J. 2017;16:53. 28137250 10.1186/s12936-017-1706-2 PMC5282846

[71] Reiter P. Climate change and mosquito-borne disease. Environ Health Perspect. 2001;109(suppl 1):141–61. 11250812 10.1289/ehp.01109s1141 PMC1240549

[72] Afrane YA, Klinkenberg E, Drechsel P, Owusu-Daaku K, Garms R, Kruppa T. Does irrigated urban agriculture influence the transmission of malaria in the city of Kumasi, Ghana? Acta Trop. 2004;89:125–34. 14732235 10.1016/j.actatropica.2003.06.001

[73] Moshi IR, Manderson L, Ngowo HS, Mlacha YP, Okumu FO, Mnyone LL. Outdoor malaria transmission risks and social life: a qualitative study in South-Eastern Tanzania. Malar J. 2018;17:397. 30373574 10.1186/s12936-018-2550-8 PMC6206631

[74] Cotter C, Sturrock HJ, Hsiang MS, Liu J, Phillips AA, Hwang J, et al. The changing epidemiology of malaria elimination: new strategies for new challenges. Lancet. 2013;382:900–11. 23594387 10.1016/S0140-6736(13)60310-4 PMC10583787

[75] Wang Z, Liu Y, Li Y, Wang G, lourençp J, Kraemer M, The relationship between rising temperatures and malaria incidence in Hainan, China, from, et al. to 2010: a longitudinal cohort study. Lancet Planet Health. 1984;2022(6):e350–8. 10.1016/S2542-5196(22)00039-0 35397223

