# Supplementary material for: Bayesian spatio-temporal modelling of environmental, climatic, and socio-economic influences on malaria in Central Vietnam
Source: Malar J. 2024 Aug 24;23:258. doi: 10.1186/s12936-024-05074-y (PMC11344946; doi:10.1186/s12936-024-05074-y)
Supplement: Supplementary file 1 — Additional file 1. [file 12936_2024_5074_MOESM1_ESM.docx]

**Supplementary Table 1.** Distribution of monthly means of environmental, climatic, and socio-economic variables, Central Vietnam, 2018–2022.

| **Variables** | **Median (IQR)** | **Min-Max** |
| --- | --- | --- |
| ALT (masl) | 291.72 (105.30 - 526.80) | 5.14 - 1339.56 |
| LSTd (ºC) | 30.70 (28.15 - 33.53) | 18.02 - 47.53 |
| LSTn (ºC) | 20.21 (18.07 - 22.26) | 5.67 - 27.48 |
| NDVI (unit) | 0.45 (0.29 - 0.56) | -0.01 - 0.78 |
| NDWI (unit) | 0.02 (0.01 - 0.04) | -0.13 - 0.12 |
| NTL (nanowatts/cm^2^/sr) | 0.50 (0.30 - 1.22) | -0.04 - 187.65 |
| PREC (mm) | 21.63 (7.71 - 43.48) | 0.06 - 298.51 |
| TMAX (ºC) | 31.38 (29.14 - 33.64) | 21.77 - 39.89 |
| TMIN (ºC) | 19.96 (17.34 - 22.23) | 6.40 - 27.33 |

*ALT* altitude, *LSTd* land surface temperature during daytime, *LSTn* land surface temperature during nighttime, *NDVI* normalized difference vegetation index, *NDWI* normalized difference water index, *NTL* nighttime lights, *PREC* precipitation, *TMAX* maximum air temperature, *TMIN* minimum air temperature, *IQR* interquartile range, *masl* meters above sea level, *mm* millimeter

**Supplementary Table 2.** Distribution of monthly means of selected environmental, climatic, and socio-economic variables for Bayesian Zero-inflated Poisson regression models of *Plasmodium falciparum* cases, Central Vietnam, 2018–2022.

| **Variables** | **Median (IQR)** | **Min-Max** |
| --- | --- | --- |
| ALT (masl) | 291.72 (105.30 - 526.80) | 5.14 - 1339.56 |
| NDVI (unit) (without lag) | 0.02 (0.01 - 0.04) | -0.13 - 0.12 |
| NTL (nanowatts/cm2 /sr)  (3-month lag) | 0.49 (0.29 - 1.20) | -0.04 - 187.65 |
| PREC (mm) (6-month lag) | 21.62 (7.64 - 41.86) | 0.06 - 298.51 |
| TMAX (ºC) (6-month lag) | 31.44 (29.19 - 33.68) | 22.64 - 39.89 |

*ALT* altitude, *NDVI* normalized difference vegetation index, *NTL* nighttime lights, *PREC* precipitation, *TMAX* maximum air temperature, *IQR* interquartile range, *masl* meters above sea level, *mm* millimeter

**Supplementary Table 3.** Distribution of monthly means of selected environmental, climatic variables for Bayesian Zero-inflated Poisson regression models of *Plasmodium vivax* cases, Central Vietnam, 2018–2022.

| **Variables** | **Median (IQR)** | **Min-Max** |
| --- | --- | --- |
| ALT (masl) | 291.72 (105.30 - 526.80) | 5.14 - 1339.56 |
| LSTd (ºC) (6-month lag) | 30.71 (28.17 - 33.53) | 10.39 - 47.53 |
| NDVI (Unit) (1-month lag) | 0.45 (0.29 - 0.56) | -0.01 - 0.78 |
| PREC (mm) (6-month lag) | 21.62 (7.64 - 41.86) | 0.06 - 298.51 |
| TMAX (ºC) (4-month lag) | 31.42 (29.19 - 33.66) | 22.64 - 39.89 |

*ALT* altitude, *LSTd* land surface temperature during daytime, *NDVI* normalized difference vegetation index, *PREC* precipitation, *TMAX* maximum air temperature, *IQR* interquartile range, *masl* meters above sea level, *mm* millimeter
